# Tuberculosis poor treatment outcomes and its determinants in Kilifi County, Kenya: a retrospective cohort study from 2012 to 2019

**DOI:** 10.1186/s13690-022-00807-4

**Published:** 2022-02-05

**Authors:** Geoffrey G. Katana, Moses Ngari, Teresia Maina, Deche Sanga, Osman A. Abdullahi

**Affiliations:** 1 Department of Public Health, Pwani University, P.O Box 195-80108, Kilifi, Kenya; 2 Kilifi County Department of Public Health, Kilifi, Kenya; 3 KEMRI Wellcome Trust Research Programme, Kilifi, Kenya; 4 Kilifi County TB Control Program, Kilifi, Kenya

**Keywords:** Tuberculosis, Poor TB treatment outcomes, Kilifi County, Retrospective study

## Abstract

**Background:**

Tuberculosis (TB) is one of the leading causes of deaths in Africa, monitoring its treatment outcome is essential to evaluate treatment effectiveness. The study aimed to evaluate proportion of poor TB treatment outcomes (PTO) and its determinants during six-months of treatment at Kilifi County, Kenya.

**Methods:**

We conducted a retrospective analysis of data from the TB surveillance system (TIBU) in Kilifi County, Kenya from 2012 to 2019. The outcome of interest was PTO (lost-to-follow-up (LTFU), death, transferred out, treatment failure, drug resistance) or successful treatment (cured or completed treatment). We performed time-stratified (at three months follow-up) survival regression analyses accounting for sub-county heterogeneity to determine factors associated with PTO.

**Results:**

We included 14,706 TB patients, their median (IQR) age was 37 (28–50) years and 8,791 (60%) were males. A total of 13,389 (91%) were on first line anti-TB treatment (2RHZE/4RH), 4,242 (29%) were HIV infected and 192 (1.3%) had other underlying medical conditions. During 78,882 person-months of follow-up, 2,408 (16%) patients had PTO: 1,074 (7.3%) deaths, 776 (5.3%) LTFU, 415 (2.8%) transferred out, 103 (0.7%) treatment failure and 30 (0.2%) multidrug resistance. The proportion of poor outcome increased from 7.9% in 2012 peaking at 2018 (22.8%) and slightly declining to 20% in 2019 (trend test *P* = 0.03). Over two-thirds 1,734 (72%) poor outcomes occurred within first three months of follow-up. In the first three months of TB treatment, overweight ((aHR 0.85 (95%CI 0.73–0.98), HIV infected not on ARVS (aHR 1.72 (95% CI 1.28–2.30)) and year of starting treatment were associated with PTO. However, in the last three months of treatment, elderly age ≥50 years (aHR 1.26 (95%CI 1.02–1.55), a retreatment patient (aHR 1.57 (95%CI 1.28–1.93), HIV infected not on ARVs (aHR 2.56 (95%CI 1.39–4.72), other underlying medical conditions (aHR 2.24 (95%CI 1.41–3.54)) and year of starting treatment were positively associated with PTO while being a female (aHR 0.83 (95%CI 0.70–0.97)) was negatively associated with PTO.

**Conclusions:**

Over two-thirds of poor outcomes occur in the first three months of TB treatment, therefore greater efforts are needed during this phase. Interventions targeting HIV infected and other underlying medical conditions, the elderly and retreated patients provide an opportunity to improve TB treatment outcome.

**Supplementary Information:**

The online version contains supplementary material available at 10.1186/s13690-022-00807-4.

## Background

Tuberculosis (TB) remains a major global public health problem and is considered one of the leading life threatening conditions [1, 2]. It ranks high above HIV/AIDS as the leading cause of morbidity and is among the top 10 causes of mortality worldwide [3]. In 2019, approximately 10 million people developed the disease and a quarter of these cases were in Africa (25%) including Kenya that ranked among the 30 high TB burden countries accounting for 21% of the global cases [3].

Currently, the control and elimination of TB is a challenge due to microbial resistance to the available drug regimen (particularly Rifampicin and Isoniazid) [4, 5]. Anti-TB drug resistance is a major medical and public health concern worldwide [6–8]. In 2019, about 61% of people with TB were tested for Rifampicin resistance and a total of 206,030 found to have MDR/RR-TB [3]. Drug resistant mycobacterium strains are major challenge facing TB control because they require longer, expensive, complex, and more toxic treatments to cure [9, 10]. Often to minimise treatment noncompliance which leads to drug resistance and improve treatment outcomes among the drug resistant TB patients directly observed treatment, short course (DOTS) programs are implemented [11, 12]. The DOTS strategy was among the three (Stop TB and End TB) scaled-up essential strategies recommended by World Health Organization (WHO) to interrupt TB transmission and the period of infectiousness [12, 13]. While the DOTS strategy has been globally adopted, TB patients still end up with poor treatment outcomes [14–17].

Treatment outcomes, as recommended by World Health Organization (WHO) in 2013, are used to assess the effectiveness of TB control programs [18]. Cured, treatment completed, treatment failed, died, and defaulted were five exclusive groups of TB treatment results used as a benchmark for global TB data collection and treatment success assessment [12]. The number of TB patients who are cured or completed treatment are considered to have a good treatment outcome, while those who missed treatment, defaulted, or died are considered to have a poor treatment outcome [19, 20]. Monitoring TB care outcomes is important for evaluating the efficacy and improvement of TB treatments, as well as identifying possible barriers to TB control [21, 22].

We identified and reviewed three systematic reviews and meta-analysis pooling the proportional of successful TB treatment and evaluating predictors of poor treatment outcomes. Torres et al. [23] reviewed 151 studies published from January 2014 to November 2019 from 59 countries representing 5 continents. The pooled treatment success rate for adults was 80.1% but was lower among the 47 studies from Africa (success rate of 78.9%). The systematic review included one study from Kenya. However, the included study was limited to smear-positive pulmonary TB patients aged 15 to 49 years only [24]. Another systematic review included 31 studies from Sub-Saharan Africa published from July 2008 to June 2018. This systematic review reported a pooled treatment success of 76.2% and did not include any study from Kenya [20]. A more recent systematic review (published 12 Oct 2021) included 26 studies from Africa. The reported pooled treatment success was 79%. Again, the systematic review did not include any study from Kenya [19]. Of interest, all the pooled treatment success rates were below the 85% reported by WHO in 2020 and lagged behind the set target of 90% TB incidence rate reduction by 2035 [25]. Predictors of poor treatment outcomes reported were HIV infection, being a retreatment patient, elderly age (>65 years) and drinking alcohol [19, 23].

In 2016, the prevalence of TB in Kenya was 558cases per 100,000 adult population with the highest burden among the adults aged 25 to 34 years (prevalence of 716 cases per 100,000 adult population) [26]. In 2019, TB was the 6th leading cause of death in Kenya [27]. The estimated prevalence of TB in Kilifi County is 122/100,000 cases according to the national survey while that of HIV is 5% [28, 29]. There is limited data on TB treatment outcomes in Kenya, specifically among TB patients in Kilifi County [19, 23, 30–32]. Therefore, the aim of this study was to estimate proportion of TB patients on treatment who end with poor outcomes and identify the determinants associated with poor treatment outcomes among tuberculosis patients in Kilifi County, Kenya.

## Methods

### Study design

This was a retrospective secondary analysis of routine standard National Leprosy and Tuberculosis and Lung Disease (NTLD) register data of Kilifi County from January 2012 to December 2019. TB treatment outcomes were categorized into successful outcomes (cured or completed six months of treatment) or poor outcomes (lost-to-follow-up, death, transferred out, treatment failure or development of drug resistance). The exposures examined were demographic (age, sex), sub-county of resident, year of starting TB treatment, nutritional status (body mass index), nutritional support provided and clinical features (HIV status, underlying comorbidities, type of TB (pulmonary or extra-pulmonary), method of TB diagnosis (bacteriological confirmed TB or empirically treated), treatment regimen and direct observed treatment.

### Setting

TB Electronic surveillance data was collected from health facilities in seven sub-counties including Kilifi North, Kilifi South, Malindi, Magarini, Kaloleni, Rabai, and Ganze in Kilifi County within the coast region of Kenya. The County had a population of 1.4 million people in 2019 census [33]. More than 70% of Kilifi County residents live in rural areas and are poor, lack formal education, and make a living from subsistence farming and fishing [34]. During the study period, there were only three health facilities with GeneXpert machines hence only ones with the capacity to diagnose TB. Not all rural health facilities have laboratory services to run sputum smear test for TB as the gold standard of TB diagnosis, nonetheless, facilities leverage the existing sputum sample referral to the high-volume health facilities for sample examination.

### Participants

The study population was all adult TB patients (≥18 years) who were on anti-TB treatment from January 2012 to December 2019 within Kilifi County.

### Variables

Pulmonary TB (PTB) was defined as any bacteriologically confirmed or clinically diagnosed case of TB involving the lung parenchyma or the tracheobronchial tree [35]. Extra-pulmonary TB (EPTB) were any bacteriologically confirmed or clinically diagnosed case of TB involving organs other than the lungs [35]. A patient was classified as transferred out if the treatment outcome was not known as a result of moving outside Kilifi County. Patients were classified as having poor treatment outcomes if they: (i) failed treatment (i.e., remaining smear-positive after 5 months of treatment), (ii) had defaulted or lost to follow-up (LTFU), (iii) died during treatment, (iv) transferred out or (v) developed drug resistance. Cured patients were those with PTB and bacteriologically confirmed at the beginning of treatment but had smear- or culture-negative test in the last one previous occasion. Deaths included all-cause mortality within the six months of follow-ups. TB patient who completed treatment without evidence of failure BUT with no record to show that sputum smear or culture results in the last month of treatment and on at least one previous occasion were negative, either because tests were not done or because results were unavailable, they were classified as having completed treatment. A TB patient whose sputum smear or culture was positive at month 5 or later during treatment was defined as treatment failure. A TB patient who did not start treatment or who started treatment but was interrupted for 2 consecutive months or more was defined as defaulted or LTFU. New TB cases were patients newly registered who had never been treated for TB before or had been on anti-TB treatment less than 4 weeks. Retreated patients were patients who had been treated for any form of TB before but had initiated treatment again following relapse, default or failure to cure after previous treatment.

### Data sources/measurements

Data were extracted from the TB Electronic surveillance system known as Treatment Information from Basic Unit (TIBU) on 10th November 2020. This system stores individual patient episodes of TB including demographic characteristics, location, clinical details, laboratory results, and treatment outcomes [36]. De-identified data were extracted directly into a Microsoft Excel spreadsheet that was designed to capture the relevant variables. Data extractions were done by GGK (first author) in presence of the County TB Coordinator.

### Study size

The study used all available eligible patient data from 2012 to 2019. A total of 14,706 patients were eligible. Assuming 14% probability of a poor outcome [37], a two-sided alpha level of 0.05, the study has power >90% to estimate a crude hazard ratio of at least 1.5 of HIV positive being associated with poor treatment outcome in the first three months of follow-up [37].

### Quantitative variables

Bacteriological confirmed TB patients were those with positive smear microscopy, culture or GeneXpert MTB/RIF result. Empirically treated patients did not have any positive TB bacteriological test but had clinical signs suggestive of TB including abnormal chest radiograph, chronic cough, fever, night sweats, weight loss, suggestive histology or extrapulmonary cases.

We created four age groups:18 to 30, 31 to 40, 41 to 50 and 51+ years. Body Mass Index (BMI) was calculated as weight (Kg) divided by square of height (meters) and further recorded into three groups according to WHO guidelines: undernourished (BMI<18.5), normal (BMI 18.5 to 25) and overweight (BMI ≥25) [38].

We assumed the missing BMI and HIV status were not missing at random. To include all patients in the regression analysis, we added extra category (missing for BMI and unknown for HIV) and used the categorical variables in the analysis. No record was missing study outcome data.

### Statistical methods

Statistical analyses were performed using STATA version 15.1 (StataCorp, College Station, TX, USA). All study patients’ characteristics were summarised using frequencies and percentages. We calculated the annual proportion of poor outcome and tested for trend across the years (from 2012 to 2019) [39].

To examine factors associated with poor treatment outcomes, we run single event survival analysis with time under observation starting from date of starting TB treatment up to 180 days later or date of any of the outcomes. All patients who completed treatment or were under treatment after 180 days were right censored at day 180. All other patients who did not complete treatment and experienced any of the poor outcomes were right censored at their last date seen alive or last follow-up. We tested the presence of heterogeneity across the seven sub-counties using likelihood ratio test in the final regression model. We found evidence for presence of sub-county heterogeneity (*P* < 0.001) and included the sub-county as random intercept in all the survival regression models using the shared frailty models [40]. We tested the Proportional-hazards (PH) assumption using the scaled Schoenfeld residuals in each independent variable and in the multivariable cox proportional hazard model with all the independent variables. Because of the violation of the PH assumption (P < 0.05), we performed time-stratified survival regression analyses. We chose to stratify the analysis at month three follow-up because this was the halfway of the follow-up time and from operational perspective, it would inform interventions targeting the early poor outcomes. However, we provided survival analyses results for the first three months and last three months of follow-up separately. We tested proportional-hazards assumption for the two time points and found no evidence of violation (the scaled Schoenfeld residuals global test for the first 3 months was *P* = 0.0936 and the last 3 months was *P* = 0.0656). We therefore used the Cox Proportional hazard regression model, running univariate model for each independent variable. To build the multivariable regression models, we used a backward stepwise approach retaining independent variables with a *P* < 0.1 and reported their hazard ratios and 95% confidence intervals. We assessed predictive values of the multivariable models using area under receiver operating curves (AUCs).

In sub-analysis, we repeated the multivariable regression models amongst bacteriological confirmed TB cases only and explored interaction between the year of starting TB treatment and various independent variables considered (age, gender, HIV status, TB diagnosis, underlying comorbidities, patient type) by comparing models with and without interaction terms using likelihood ratio test.

## Results

### Patient characteristics

**Fig. 1:**
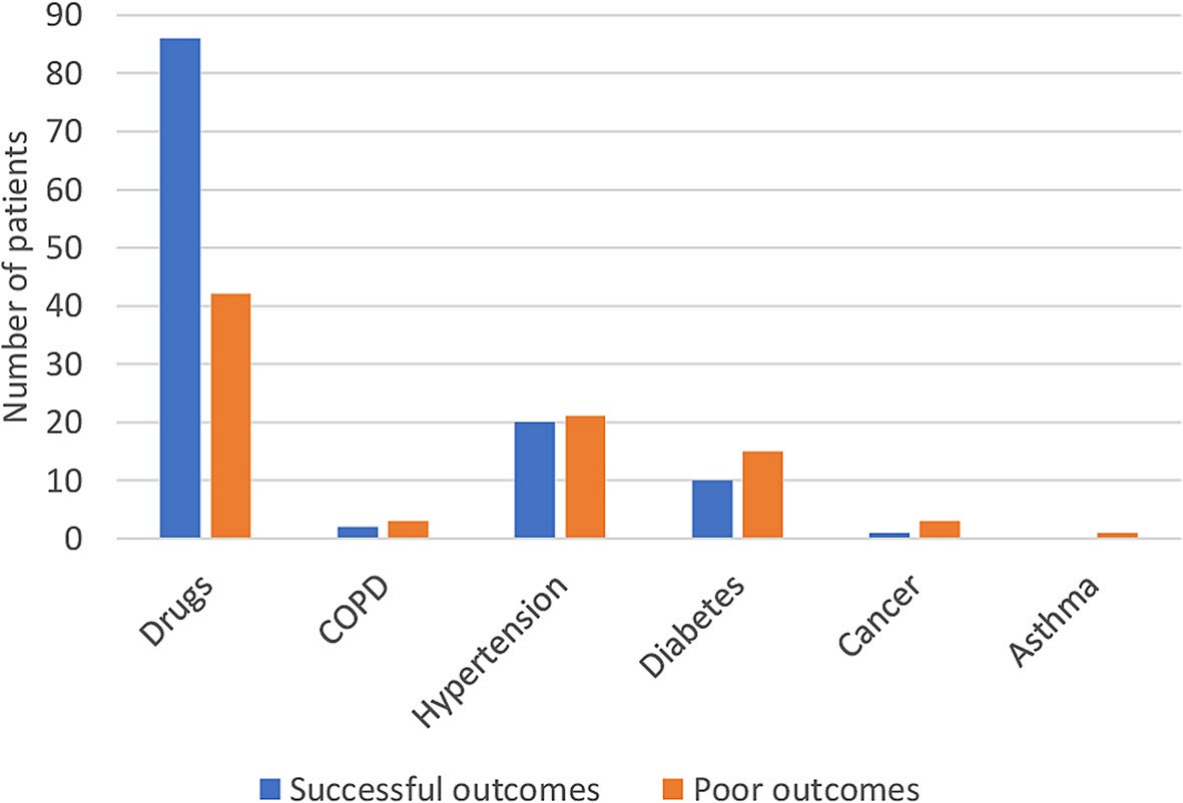
List of underlying comorbidities among patients on TB treatment at Kilifi County from 2012 to 2019. COPD; chronic obstructive pulmonary disease, a patient could have more than one comorbidity

During the study period, 14,706 patients were started on anti-TB treatment. Their median (Interquartile range (IQR)) age was 37 (28 to 50) years and 8,791 (60%) were male. About 13,027 (89%) were new TB patients while 12,975 (88%) had (PTB) and 1,731 (11%) had EPTB. Approximately half: 51% patients had normal BMI while 4,309 (29%) were undernourished. About 4,242 (29%) were HIV infected, of which 4,037/4,242 (95%) were on ARVs and 4,212/4,242 (99%) were on cotrimoxazole prophylaxis. More than three quarters: 78% of the patients were treated in a public health facility while 13,100 (89%) were on family-based direct observation treatment. A total of 13,389 (91%) patients were on Rifampicin (R), Isoniazid (H), Pyrazinamide (Z), Ethambutol (E) for the first two months and Rifampicin (R), Isoniazid (H) for the following four months (2RHZE/4RH) TB treatment regimen (Table 1). Approximately half of the patients (*N* = 7,293, 50%) were started on anti-TB empirically based on clinical signs. One hundred and ninety-two (1.3%) patients had underlying comorbidities: 128 (0.9%) were taking drugs (smoking for more than six months, drinking alcohol etc.), 5 (0.03%) had chronic obstructive pulmonary disease (COPD), 41 (0.3%) had hypertension, 25 (0.2%) had diabetes, 4 (0.03%) had cancer and 1 (0.01%) had asthma (Fig. 1).

**Table 1:** Patients characteristics when starting TB treatment at Kilifi County from 2012 to 2019

Features	Successful outcomes (*N* = 12,298)	Poor outcomes (*N* = 2408)	All patients (*N* = 14,706)
Sex			
Male	7265 (59)	1526 (63)	8791 (60)
Female	5033 (41)	882 (37)	5915 (40)
Age in years			
18 to 30 years	4097 (33)	692 (29)	4789 (33)
31 to 40 years	3397 (28)	602 (25)	3999 (27)
41 to 50 years	2067 (17)	423 (18)	2490 (17)
51 + years	2737 (22)	691 (29)	3428 (23)
Patient type			
New cases	10,953 (89)	2074 (86)	13,027 (89)
Re-treatment cases	1345 (11)	334 (14)	1679 (11)
TB type			
Pulmonary	10,875 (88)	2100 (87)	12,975 (88)
Extrapulmonary	1423 (12)	308 (13)	1731 (12)
Nutrition status			
Undernourished	3582 (29)	727 (30)	4309 (29)
Normal BMI	6360 (52)	1185 (49)	7545 (51)
Overweight	1588 (13)	290 (12)	1878 (13)
Missing	768 (6.2)	206 (8.6)	974 (6.6)
HIV status			
HIV uninfected	8827 (72)	1484 (62)	10,311 (70)
HIV infected on ARVS	3203 (26)	834 (35)	4037 (27)
HIV infected not on ARVS	144 (1.2)	61 (2.5)	205 (1.4)
Unknown HIV status	124 (1.0)	29 (1.2)	153 (1.0)
Sector of recruitment health facility			
Public	9669 (79)	1852 (77)	11,521 (78)
Private	2402 (20)	527 (22)	2929 (20)
Prisons	227 (1.9)	29 (1.2)	256 (1.7)
DOT			
Family-based	10,939 (89)	2161 (90)	13,100 (89)
Community volunteer	717 (5.8)	121 (5.0)	838 (5.7)
Health worker	642 (5.2)	126 (5.2)	768 (5.3)
Treatment regimen			
2RHZE/4RH	11,223 (91)	2166 (90)	13,389 (91)
2SRHZE/1RHZE/5RHE	881 (7.2)	199 (8.3)	1080 (7.3)
2RHZ/4RH	137 (1.1)	31 (1.3)	168 (1.2)
Others	57 (0.5)	12 (0.5)	69 (0.5)
TB diagnosis			
Bacteriologically confirmed	6262 (51)	1151 (48)	7413 (50)
Clinical signs	6036 (49)	1257 (52)	7293 (50)
Nutritional support			
No support	110 (0.9)	28 (1.2)	138 (0.9)
Nutritional counselling	125 (1.0)	17 (0.7)	142 (1.0)
Counselling & food support	8430 (69)	1818 (75)	10,248 (70)
Food support & no counselling	3633 (30)	545 (23)	4178 (28)
Underlying comorbidities	113 (0.9)	79 (3.3)	192 (1.3)
Sub County			
Kilifi North	2364 (19)	492 (20)	2856 (19)
Kilifi South	1996 (16)	392 (16)	2388 (16)
Kaloleni	2317 (19)	524 (22)	2841 (19)
Malindi	2905 (24)	609 (25)	3514 (24)
Magarini	1451 (12)	202 (8.4)	1653 (11)
Ganze	632 (5.1)	93 (3.9)	725 (4.9)
Rabai	633 (5.2)	96 (4.0)	729 (5.0)

*DOT* Direct observed treatment, *BMI *Body Mass Index, *ARVs *Antiretroviral

### Outcome

**Fig. 2:**
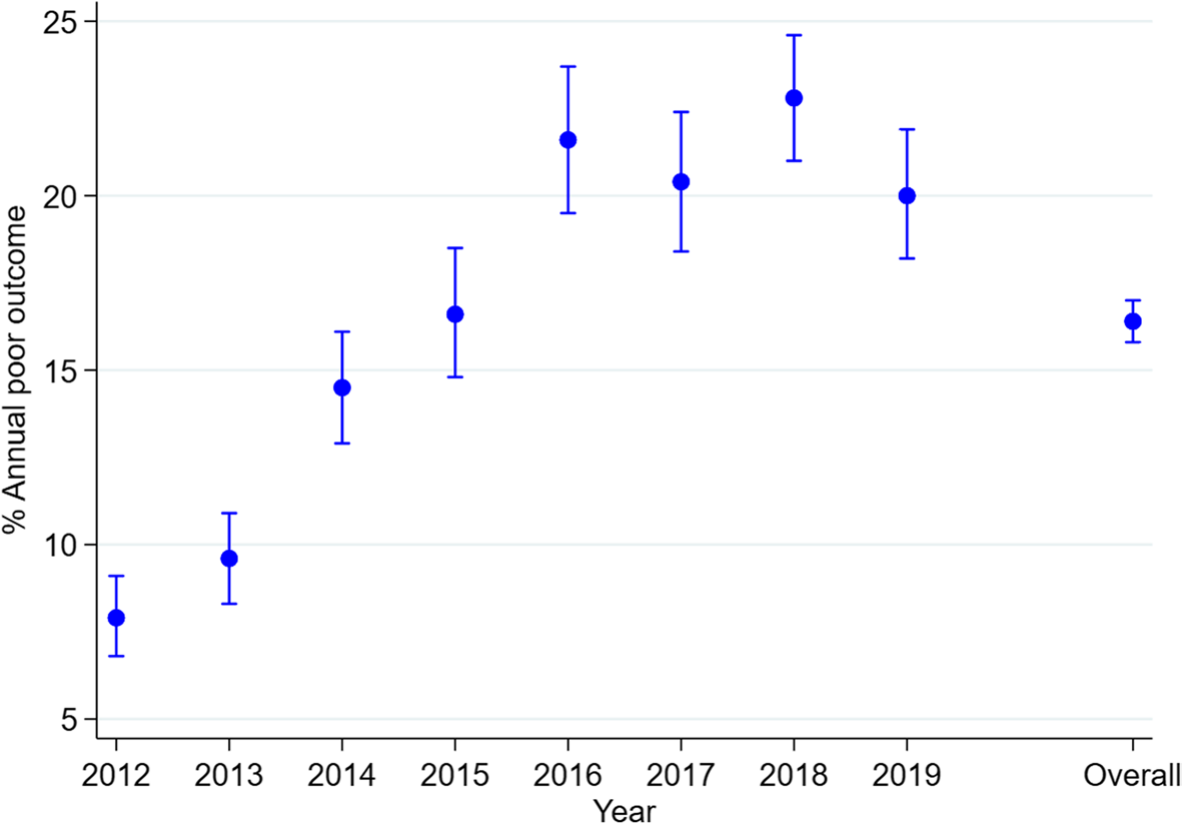
Trend in annual proportion of poor outcomes among patients on TB treatment at Kilifi County from 2012 to 2019. Trend *p*-value=0.03

Of 14,706 patients on treatment, 2,408 had poor outcomes (16.3%, (95% CI 15.8 to 17.0)). The 2,408 poor outcomes were: deaths (*n* = 1,074, 7.3%), LTFU (*n* = 776, 5.3%), transferred out (*n* = 425, 2.8%), treatment failure (*n* = 103, 0.7%) and multidrug resistance (*n* = 30, 0.2%). The proportion of poor outcome increased from 7.9% in 2012 peaking at 2018 (22.8%) and slightly declining to 20% in 2019 (test for trend *P* = 0.03) Fig. 2.

### Follow-up time

The patients were on follow-up for a total of 78,882 person-months: 73,788 and 5,094 person-months among the successful and poor outcomes patients respectively. The poor outcome incidence rate was 472 (95% CI 454–491) per 1,000 person-months. The patient with poor treatment outcomes were on follow-up for median (IQR) 55 (51 to 58) days. Of the 2,408 patients with poor treatment outcomes, 117 (4.9%) occurred on the day of starting TB treatment (70/117 were LTFU, 14/117 died and 33/117 transferred out). Most poor outcomes occurred early: 741/2,408 (31%), 1,282/2,408 (53%) and 1,734/2,408 (72%) occurring within the first one, two and three months respectively.

### Factors associated with poor outcomes

In the univariate model of the first three months of TB treatment, HIV infected not on ARVs (Crude Hazard Ratio (CHR) 1.59 (95%CI 1.19–2.11)), patients empirically treated without bacteriological confirmation (CHR 1.16 (95%CI 1.05–1.28)) and the year of starting treatment were significantly associated with higher hazard of poor outcomes. However, overweight (CHR 0.84 (95%CI 0.72–0.98)) was negatively associated with hazard of poor outcome (Additional file 1).

In the univariate model of the last three months of TB treatment, elderly age (≥ 51years) (CHR 1.30 (95%CI 1.06–1.59)), a retreatment patient (CHR 1.69 (95%CI 1.38–2.06)), HIV infected on ARVs (CHR 1.48 (95%CI 1.26–1.73)), 2SRHZE/1RHZE/5RHE treatment regimen, underlying conditions and year of starting treatment were significantly associated with higher hazard of poor outcomes. However, females (CHR 0.84 (95%CI 0.71–0.98)) and patients empirically treated without bacteriological confirmation (CHR 0.81 (95%CI 0.69–0.94)) were negatively associated with hazard of poor outcomes (Additional file 1).

**Fig. 3:**
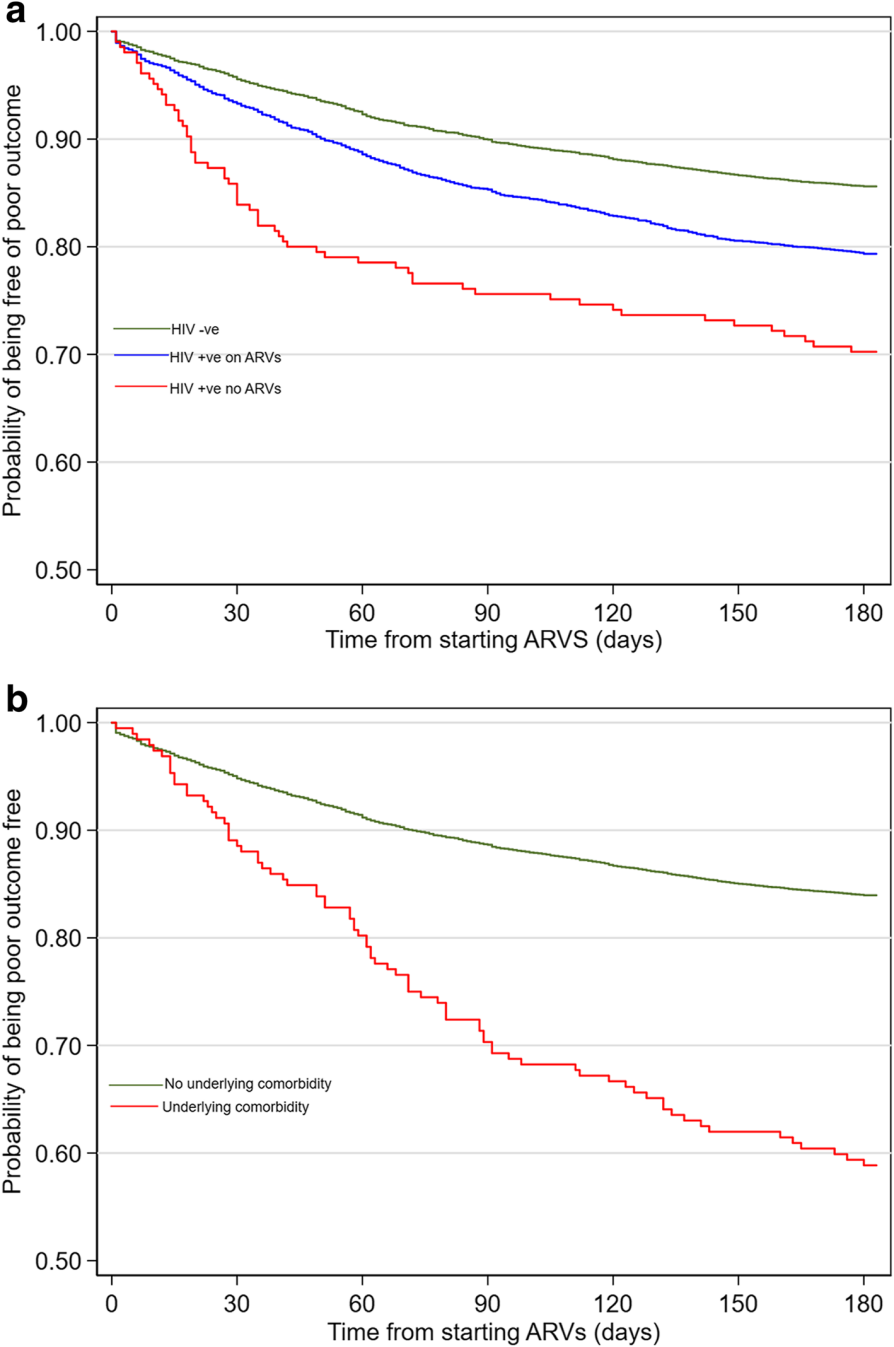
Kaplan-Meier plot of time to poor outcome by: (**a**) HIV status and (**b**) underlying comorbidity among patients on TB treatment at Kilifi County from 2012 to 2019

In the multivariable regression model of the first three months of TB treatment, HIV infected not on ARVs (adjusted Hazard Ratio (aHR) 1.72 (95%CI 1.28–2.30)) (Fig. 3a) and the year of starting treatment were significantly associated with higher hazard of poor outcomes. Being overweight (aHR 0.85 (95%CI 0.73–0.98)) was negatively associated with hazard of poor outcome (Table 2). We found evidence of interaction between being overweight and type of health facility (*P* = 0.02). Overweight was more common among patients recruited from private health facilities (14%) compared to those recruited from public health facilities (12%) and from prisoners (9.7%). The multivariable model AUC (95%CI) was 0.60 (95%CI 0.58–0.62).

**Table 2:** Multivariable analysis of factors associated with poor TB treatment outcomes at Kilifi County from 2012 to 2019

	First 3 months		Last 3 months	
	Adjusted HR (95%CI)	*P*-value	Adjusted HR (95%CI)	*P*-value
Sex				
Male	^a^		Reference	
Female	^a^		0.83 (0.70–0.97)	0.02
Age in years				
18 to 30 years	^a^		Reference	
31 to 40 years	^a^		0.97 (0.80–1.19)	0.80
41 to 50 years	^a^		0.95 (0.75–1.20)	0.66
51 + years	^a^		1.26 (1.02–1.55)	0.03
Patient type				
New cases	^a^		Reference	
Re-treatment cases	^a^		1.57 (1.28–1.93)	<0.001
Nutrition status				
Undernourished	0.96 (0.86–1.07)	0.47	^a^	
Normal BMI	Reference		^a^	
Overweight	0.85 (0.73–0.98)	0.04	^a^	
HIV status				
HIV uninfected	Reference		Reference	
HIV infected on ARVS	1.08 (0.98–1.20)	0.13	1.65 (1.39–1.96)	<0.001
HIV infected not on ARVS	1.72 (1.28–2.30)	<0.001	2.56 (1.39–4.72)	0.003
Unknown HIV status	1.12 (0.74–1.68)	0.60	0.92 (0.34–2.46)	0.86
Underlying conditions	^a^		2.24 (1.41–3.54)	0.001
TB diagnosis				
Bacteriologically confirmed	^a^		Reference	
Clinical signs	^a^		0.82 (0.70–0.97)	0.02
Year of starting treatment				
2012	Reference		Reference	
2013	1.56 (1.20–2.02)	0.001	1.37 (0.95–1.98)	0.10
2014	1.55 (1.22–1.96)	<0.001	1.67 (1.16–2.40)	0.005
2015	1.58 (1.24–2.00)	<0.001	2.24 (1.57–3.20)	<0.001
2016	1.40 (1.11–1.76)	0.004	3.06 (2.17–4.31)	<0.001
2017	1.79 (1.42–2.25)	<0.001	2.80 (1.99–3.94)	<0.001
2018	1.93 (1.55–2.39)	<0.001	2.48 (1.77–3.47)	<0.001
2019	1.59 (1.27–1.99)	<0.001	2.30 (1.62–3.27)	<0.001
AUCs (95% CI)	0.60 (0.58–0.62)		0.64 (0.63–0.65)	

^a^variables were not selected for inclusion in multivariable model

In the multivariable regression model of the last three months of TB treatment, elderly age (≥ 51years) (aHR 1.26 (95%CI 1.02–1.55)), a retreatment patient (aHR 1.57 (95%CI 1.28–1.93)), HIV infected on ARVs and not on ARVs, underlying comorbidities (aHR 2.24 (95%CI 1.41–3.54)) **(**Fig. 3b) and year of starting treatment were significantly associated with higher hazard of poor outcomes. Being female (aHR 0.83 (95%CI 0.70–0.97)) and patients empirically treated without bacteriological confirmation (aHR 0.82 (95%CI 0.70–0.97)) were negatively associated with hazard of poor outcome (Table 2). The multivariable regression model AUC was 0.64 (95%CI 0.63–0.65).

### Sub-analysis

In the sub-analysis, factors associated with poor outcomes in the multivariable regression models in the first and last three months of treatment including only TB confirmed cases, were approximately similar to the whole population (Additional file 2). We found no evidence of interaction between year of starting TB treatment and age (*P *= 0.56), sex (*P *= 0.62), HIV status (*P *= 0.70), type of TB diagnosis (*P *= 0.32), underlying comorbidities (*P *= 0.13) and patient type (*P *= 0.28) in the first three months of TB treatment. Similarly, there was no interaction between year of starting TB treatment and age (*P *= 0.54), sex (*P *= 0.92), HIV status (*P *= 0.15), type of TB diagnosis (*P *= 0.11), underlying comorbidities (*P *= 0.63) and patient type (*P *= 0.42) in the last three months of TB treatment.

## Discussion

In this large study including >14,000 participants, poor treatment outcomes frequently (more than two thirds) occurred very early after starting TB treatment usually within the first three months. Characteristics of the patients with poor outcomes in the first three months and after three months were different suggesting different strategies to improve early and late treatment outcomes are needed. The 16% poor outcome in our study was similar with the findings reported in Southern Ethiopia, Somalia, India, and Russia [41–44]. However, our prevalence was higher than other studies in China (4.2%) and Europe (12.5%) [45, 46]. The low prevalence of poor outcome in China and Europe could be attributable to their more responsive health systems.

In the early phase of treatment where more than two-thirds of poor outcomes occurred, being HIV infected not on ARVs was associated with higher risk of poor outcome. This is an important phase of TB treatment where majority of the patients were on four drugs for the first two months and two drugs for the last four months [47]. The extra burden of taking ARVs and cotrimoxazole prophylaxis by the HIV infected patients and possible drug interactions with adverse effects might have negatively affected the patients impairing their TB treatment outcomes [48, 49]. TB and HIV diagnosis have also been associated with stigma, which might further have adverse effects on TB treatment outcomes [50, 51]. Like our study, a number of previous studies have found HIV association with poor TB treatment outcomes [52–54]. Surveillance systems like the one in place in our setting should play a key role in providing data for action. Owing to limited resources, our surveillance system is not able to actively monitor the patients in the community. However, evidence demonstrates simple and cheap strategies including digital technology (like reminders via short message services (SMSs) through mobile phones), use of community health volunteers to offer patient education and psychological support improves TB treatment outcomes [55, 56]. Therefore, our findings of poor outcomes majorly among the HIV coinfected patients should trigger the surveillance managers to explore strategies of integrating retention methods with the ones provided by HIV programs and possible adoption of cheap strategies like reminders via digital technology. This finding also highlights the need for systematic HIV screening among all presumptive TB patients and prompt treatment for those who test positive.

In the last three months of treatment, patients on retreatment, the elderly and those with underlying medical conditions including HIV were associated with poor outcomes. It is likely that patients on retreatment had poor outcomes because they developed drug resistant strains as reported elsewhere [57]. Also, studies show that patient’s behaviour influences unsuccessful treatment [30, 58]; a study looking at factors associated with poor outcomes indicated that patients who get lost to follow-up who then have to be retreated for TB are often reluctant to uptake and tend to interrupt treatment [41]. Studies conducted elsewhere have also shown the risk of poor TB treatment outcomes increases as age advances because old age comes with increased age-related immunosuppressant comorbidities such as diabetes mellitus that increase adverse effects of anti-TB drugs, cause drug resistance, mortality, and increases recurrence of TB in this group [49, 59, 60]. In addition to retreatment and old age complexities, underlying conditions such as HIV worsens treatment outcomes as reported in other studies by synergistically interacting with TB to alter its clinical manifestation, complicate the treatment follow-up process, and to cause death [61–64]. This finding suggests the need for strategies targeting patients with these features as a `*high risk*’ group for poor treatment outcome.

In the six months of follow-up, poor treatment outcome increased over the years. We found no evidence of interaction between the age of starting treatment and other exposures like HIV or age. Previous research in this cohort of patients had similar findings suggesting deteriorating TB treatment outcomes over years [37, 65]. This is a very worrying trend requiring further research. We hypothesis this could be driven by conditions such as diabetes mellitus that increase adverse effects of anti-TB drugs, cause drug resistance, mortality, and increases recurrence of TB in this group [66–68]. Given the increasing prevalence of diabetes mellitus in regions with a high TB burden, there is need for TB control programs to closely monitor and treat patients presenting with diabetes mellitus for improved treatment outcomes to be achieved [68].

Future studies should focus on factors not explored or collected in this study. Qualitative studies exploring patients experience with TB treatment and interaction with health workers every month would provide an opportunity for an in-depth understanding of the barriers to treatment success.

### Study strengths and limitations

The main strength of the study is the large size and the robust analysis conducted. Being surveillance, our study was limited to the available data. We therefore did not have access to other variables such as other comorbidities and behavioural and socio-economic factors (like alcohol consumption, smoking, income, living conditions, education, and family size) which might affect treatment outcomes. The surveillance system lacks resources to implement active surveillance, thus the high proportion of TB poor treatment outcomes may not be generalizable in settings with resources to support active surveillance.

## Conclusions

Poor treatment outcomes more frequently occur in the first three months following initiation of TB treatment and therefore greater efforts are needed during this phase. Our study findings suggest the need for different strategies to improve TB treatment outcomes during the first and last three months of treatment. Strategies targeting the elderly, retreated patients, HIV infected and those with underlying medical conditions provide an opportunity to improve TB treatment outcomes. The TB program team needs to offer more support to reverse the increasing annual poor TB treatment outcome trend.

## Supplementary Information

**Additional file 1.**

**Additional file 2.**

## Data Availability

The study data are available from the corresponding author on reasonable request.

## Abbreviations

TB: Tuberculosis
PTO: Poor TB Treatment Outcome
IQR: Inter-quartile Range
HIV: Human Immunodeficiency Virus
LTFU: Lost to Follow-up
AIDS: Acquired Immunodeficiency Syndrome
MDR/RR-TB: Multidrug- and Rifampicin-Resistant Tuberculosis
DOTS: Directly Observed Treatment Short Course
WHO: World Health Organization
NTLD: National Leprosy and Tuberculosis and Lung Disease
PTB: Pulmonary TB
EPTB: Extra-pulmonary TB
TIBU: Treatment Information from Basic Unit
BMI: Body Mass Index
AUC: Area Under the Curve
RECORD: Reporting of studies Conducted using Observational Routinely-collected health Data
ARVs: Antiretroviral Drugs
COPD: Chronic Obstructive Pulmonary Disease
SMSs: Short Message Services
