# Supplementary material for: Tuberculosis poor treatment outcomes and its determinants in Kilifi County, Kenya: a retrospective cohort study from 2012 to 2019
Source: Arch Public Health. 2022 Feb 5;80:48. doi: 10.1186/s13690-022-00807-4 (PMC8818215; doi:10.1186/s13690-022-00807-4)
Supplement: Supplementary file 1 — Additional file 1. [file 13690_2022_807_MOESM1_ESM.docx]

Additional file 1. **Univariate analysis of factors associated with poor TB treatment outcomes at Kilifi County from 2012 to 2019.**

|  | First 3 months | | Last 3 months | |
| --- | --- | --- | --- | --- |
|  | Crude HR (95%CI) | P-value | Crude HR (95%CI) | P-value |
| Sex |  |  |  |  |
| Male | Reference |  | Reference |  |
| Female | 1.06 (0.96–1.17) | 0.22 | 0.84 (0.71–0.98) | 0.03 |
| Age in years |  |  |  |  |
| 18 to 30 years | Reference |  | Reference |  |
| 31 to 40 years | 1.06 (0.93–1.21) | 0.37 | 1.11 (0.91–1.34) | 0.31 |
| 41 to 50 years | 1.04 (0.91–1.21) | 0.55 | 1.13 (0.90–1.42) | 0.31 |
| 51 + years | 1.13 (1.00–1.29) | 0.05 | 1.30 (1.06–1.59) | 0.01 |
| Patient type |  |  |  |  |
| New cases | Reference |  | Reference |  |
| Re-treatment cases | 0.90 (0.78–1.04) | 0.15 | 1.69 (1.38–2.06) | <0.001 |
| TB type |  |  |  |  |
| Pulmonary | Reference |  | Reference |  |
| Extrapulmonary | 1.14 (0.99–1.31) | 0.07 | 1.01 (0.79–1.27) | 0.97 |
| Nutrition status |  |  |  |  |
| Undernourished | 0.95 (0.85–1.06) | 0.36 | 1.14 (0.96–1.36) | 0.14 |
| Normal BMI | Reference |  | Reference |  |
| Overweight | 0.84 (0.72–0.98) | 0.03 | 0.96 (0.75–1.23) | 0.74 |
| HIV status |  |  |  |  |
| HIV uninfected | Reference |  | Reference |  |
| HIV infected on ARVS | 1.09 (0.98–1.20) | 0.10 | 1.48 (1.26–1.73) | <0.001 |
| HIV infected not on ARVS | 1.59 (1.19–2.11) | 0.002 | 1.54 (0.84–2.80) | 0.16 |
| Unknown HIV status | 1.18 (0.79–1.76) | 0.41 | 0.68 (0.25–1.81) | 0.44 |
| Sector of recruitment health facility |  |  |  |  |
| Public | Reference |  | Reference |  |
| Private | 0.96 (0.86–1.08) | 0.51 | 1.08 (0.89–1.31) | 0.43 |
| Prisons | 0.74 (0.48–1.15) | 0.18 | 0.64 (0.30–1.37) | 0.25 |
| DOT |  |  |  |  |
| Family-based | Reference |  | Reference |  |
| Community volunteer | 0.86 (0.68–1.08) | 0.18 | 1.02 (0.74–1.41) | 0.90 |
| Health worker | 0.94 (0.75–1.18) | 0.60 | 1.24 (0.91–1.69) | 0.17 |
| Treatment regimen |  |  |  |  |
| 2RHZE/4RH | Reference |  | Reference |  |
| 2SRHZE/1RHZE/5RHE | 0.87 (0.73–1.04) | 0.12 | 1.42 (1.10–1.84) | 0.007 |
| 2RHZ/4RH | 1.16 (0.74–1.80) | 0.52 | 1.4f8 (0.81–2.68) | 0.20 |
| Others | 1.49 (0.62–3.59) | 0.38 | 2.18 (1.03–4.59) | 0.04 |
| Nutritional support |  |  |  |  |
| No support | 1.01 (0.65–1.57) | 0.96 | 1.20 (0.60–2.42) | 0.61 |
| Nutritional counselling | 0.82 (0.48–1.39) | 0.46 | 0.40 (0.13–1.26) | 0.12 |
| Counselling & food support | Reference |  | Reference |  |
| Food support & no counselling | 0.90 (0.80–1.02) | 0.10 | 0.71 (0.59–0.86) | <0.001 |
| Underlying conditions | 0.80 (0.62–1.05) | 0.10 | 2.99 (1.91–4.69) | <0.001 |
| TB diagnosis |  |  |  |  |
| Bacteriologically confirmed | Reference |  | Reference |  |
| Clinical signs | 1.16 (1.05–1.28) | 0.004 | 0.81 (0.69–0.94) | 0.006 |
| Year of starting treatment |  |  |  |  |
| 2012 | Reference |  | Reference |  |
| 2013 | 1.66 (1.28–2.14) | <0.001 | 1.40 (0.97–2.02) | 0.08 |
| 2014 | 1.65 (1.31–2.08) | <0.001 | 1.69 (1.18–2.42) | 0.004 |
| 2015 | 1.55 (1.23–1.96) | <0.001 | 2.29 (1.61–3.26) | <0.001 |
| 2016 | 1.39 (1.11–1.75) | 0.004 | 3.15 (2.24–4.42) | <0.001 |
| 2017 | 1.73 (1.38–2.17) | <0.001 | 2.92 (2.08–4.10) | <0.001 |
| 2018 | 1.91 (1.54–2.37) | <0.001 | 2.61 (1.87–3.64) | <0.001 |
| 2019 | 1.55 (1.24–1.93) | <0.001 | 2.47 (1.75–3.49) | <0.001 |
