# Supplementary material for: Tuberculosis poor treatment outcomes and its determinants in Kilifi County, Kenya: a retrospective cohort study from 2012 to 2019
Source: Arch Public Health. 2022 Feb 5;80:48. doi: 10.1186/s13690-022-00807-4 (PMC8818215; doi:10.1186/s13690-022-00807-4)
Supplement: Supplementary file 2 — Additional file 2. [file 13690_2022_807_MOESM2_ESM.docx]

Additional file 2. **Multivariable sub-analysis of factors associated with poor TB treatment outcomes among TB confirmed cases at Kilifi County from 2012 to 2019.**

|  | First 3 months | | Last 3 months | |
| --- | --- | --- | --- | --- |
|  | Adjusted HR (95%CI) | P-value | Adjusted HR (95%CI) | P-value |
| Sex |  |  |  |  |
| Male | ‡ |  | Reference |  |
| Female | ‡ |  | 0.75 (0.60–0.94) | 0.01 |
| Age in years |  |  |  |  |
| 18 to 30 years | Reference |  | Reference |  |
| 31 to 40 years | 1.02 (0.85–1.22) | 0.85 |  |  |
| 41 to 50 years | 1.10 (0.87–1.38) | 0.44 |  |  |
| 51 + years | 1.21 (0.99–1.49) | 0.06 |  |  |
| Patient type |  |  |  |  |
| New cases | ‡ |  | Reference |  |
| Re-treatment cases | ‡ |  | 1.63 (1.25–2.13) | <0.001 |
| HIV status |  |  |  |  |
| HIV uninfected | Reference |  | Reference |  |
| HIV infected on ARVS | 1.14 (0.97–1.33) | 0.12 | 1.38 (1.09–1.74) | 0.007 |
| HIV infected not on ARVS | 2.65 (1.66–4.23) | <0.001 | 3.76 (1.75–8.12) | 0.001 |
| Unknown HIV status | 1.37 (0.74–2.53) | 0.32 | 1.35 (0.43–4.23) | 0.61 |
| Underlying conditions | 0.66 (0.46–0.95) | 0.02 | 2.00 (1.13–3.55) | 0.02 |
| Year of starting treatment |  |  |  |  |
| 2012 | Reference |  | Reference |  |
| 2013 | 1.57 (1.03–2.40) | 0.04 | 1.17 (0.65–2.12) | 0.61 |
| 2014 | 1.29 (0.88–1.89) | 0.19 | 2.11 (1.23–3.62) | 0.007 |
| 2015 | 1.31 (0.93–1.83) | 0.12 | 2.62 (1.56–4.41) | <0.001 |
| 2016 | 1.19 (0.86–1.65) | 0.30 | 3.82 (2.31–6.31) | <0.001 |
| 2017 | 1.49 (1.06–2.07) | 0.02 | 3.24 (1.95–5.38) | <0.001 |
| 2018 | 1.78 (1.28–2.48) | 0.001 | 2.67 (1.59–4.49) | <0.001 |
| 2019 | 1.36 (0.98–1.90) | 0.06 | 3.33 (2.01–5.53) | <0.001 |
| ‡variables were not selected for inclusion in multivariable model. | | | | |
